# The performance of two-implant overdentures in the atrophic maxilla: a case series with 1-year follow-up

**DOI:** 10.1186/s40729-022-00460-0

**Published:** 2022-12-09

**Authors:** Pieter Onclin, Caroline M. Speksnijder, Henny J. A. Meijer, Arjan Vissink, Gerry M. Raghoebar

**Affiliations:** 1 Department of Oral and Maxillofacial Surgery, University Medical Center Groningen and University of Groningen, PO Box 30.001, 9700 RB Groningen, The Netherlands; 2 Department of Oral-Maxillofacial Surgery, Prosthodontics and Special Dental Care, University Medical Center Utrecht, Utrecht University, Utrecht, The Netherlands; 3 Department of Implant Dentistry, University of Groningen and University Medical Center Groningen, Groningen, The Netherlands

**Keywords:** Edentulous, Atrophic, Maxilla, Implant, Overdenture, Masticatory performance

## Abstract

**Purpose:**

To assess the implant and prosthesis survival rates, the clinical, radiographical and patient-related outcome measures, and the masticatory performance of maxillary overdentures supported by two implants in patients with an atrophic maxilla.

**Methods:**

In this case series, 15 consecutive patients who were eligible for maxillary implant overdenture therapy, but who had insufficient bone volume to place at least four implants and were unwilling to be treated with reconstructive surgery were asked to participate. After giving consent, participants received two implants in the maxilla under local anaesthesia. After 3 months of osseointegration, a maxillary overdenture with palatal coverage and solitary attachments was fabricated. Implant and overdenture survival, marginal bone level change, clinical outcome measures, masticatory performance and patient-related outcomes were evaluated at baseline and 1 year after overdenture placement.

**Results:**

Fourteen out of 15 participants completed the follow-up period of 12 months. Implant and overdenture survival rate were 89.3% and 85.7%, respectively. Change in marginal bone level (− 0.5 ± 0.7 mm), change in probing depth (0.0 ± 1.0 mm), and clinical outcomes were favourable. Masticatory performance and patient-related outcomes improved significantly compared to baseline. Complications were minimal.

**Conclusions:**

Within the limitations of this study, it can be concluded that patients with extreme resorption of the maxilla that are unwilling to be treated with reconstructive surgery, benefit from two-implant maxillary overdentures retained by solitary attachments in terms of improved masticatory functioning and denture satisfaction. However, they have relatively high risk of implant loss.

*Trial registration:* UMCG Trial Register (RR201900060), registered 22 January 2019.

**Graphical Abstract:**

## Background

Though there is an ongoing debate on the number of implants and the preferable superstructure in the edentulous maxilla [1], an implant overdenture (IOD) retained by four implants is a good treatment option for patients experiencing problems with their conventional maxillary denture [2–4]. After a follow-up of at least 5 years, marginal bone level change (MBLC), implant and overdenture survival, and clinical parameters such as probing depth change (PDC), plaque and bleeding on probing are favourable, mastication is improved, and patient satisfaction is high [2–5].

In case of extreme bone resorption, where no reliable implant placement is possible, reconstructive surgery using bone augmentation may be needed or zygomatic implants can be used. Both therapies are reliable and safe, but can be invasive, can induce some morbidity and due to its extent it is often performed under general anaesthesia [6]. A maxillary IOD can also be supported by less than four implants. Placing less than four implants to support a maxillary IOD may avoid the need for reconstructive surgery, is less invasive and less time-consuming and enables implant placement in an outpatient treatment setting. However, current evidence of such treatment is still sparse and show variable clinical, radiographical and patient-related outcomes [7–11]. With an increasingly aging population and subsequent age-associated comorbidities, the demand for less invasive elective surgical procedures is increasing. Therefore, the purpose of this case series was to assess the implant survival rate (primary outcome), prosthesis survival rates, the clinical, radiographical and patient-related outcome measures, and the masticatory performance (secondary outcomes) of maxillary overdentures supported by two implants in patients with an atrophic maxilla. The null-hypothesis was: a two-implant maxillary overdenture on solitary attachments performs well with respect to implant survival, clinical, radiographical and patient-related outcome measures of patients with extreme resorption of the maxilla that are unwilling to be treated with reconstructive surgery.

## Methods

Between September 2017 and September 2020 all patients experiencing problems with their maxillary overdenture that were referred to the Department of Oral and Maxillofacial Surgery of the University Medical Center Groningen in Groningen, the Netherlands were screened if they were eligible for maxillary implant overdenture therapy. Patients that had insufficient bone volume to place at least four implants in the edentulous maxilla and were unwilling to be treated with reconstructive surgery, were asked to participate in this case series. To be able to participate, the bone volume in the anterior maxilla, as assessed on a cone beam computed tomography (CBCT), had to be sufficient for the placement of two implants. A participant had to be at least 18 years of age, fully edentulous for at least 1 year and did not have an American Society of Anesthesiologists score (ASA) of IV or higher [12].

In this study the participants were treated following an existing procedure and was, therefore, not considered research performed on test-subjects as meant in the Medical Research Involving Human Subjects Act (WMO) (MEC-reference M19.224998). The study was registered in the UMCG Trial Register (RR201900060). This study was conducted in accordance with the 2008 revised requirements of the Helsinki Declaration of 1975.

### Surgical procedure

**Fig. 1:**
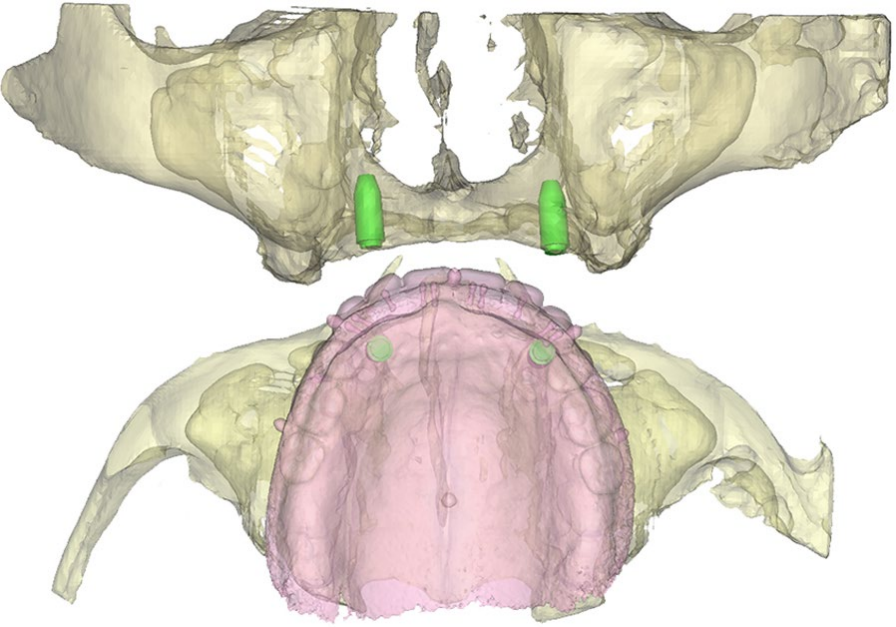
3D planning of the implant locations

All the implants were planned in the canine region using 3D Virtual Surgical Planning using computer software (Proplan CMF software; Materialise, Leuven, Belgium) to ensure an optimised implant location from both a surgical and prosthetic perspective. The implant positions were transferred to surgical template using computer software (3-Matic Medical 11.0; Materialise, Leuven, Belgium; Fig. 1).

All the participants were treated under local anaesthesia (Ultracain® D-S forte, Sanofi Aventis, Gouda, Netherlands) by the same oral and maxillofacial surgeon (GMR). The implants were placed using the surgical template and consecutive diameter drill sleeves, following the manufacturer’s instructions. After removal of the template, two implants (Nobel Active NP 3.5 mm, Nobel Biocare®, Zurich, Switzerland) were placed with a minimum torque of 45 Ncm. Small bone dehiscences were covered with intra-orally harvested bone and a resorbable membrane (Bio-Gide®, Geistlich Pharma North America Inc., Princeton, USA). After the insertion of cover screws, the flap was repositioned and sutured. All participants received antibiotics (500 mg Clamoxyl, GlaxoSmithKline, Utrecht, the Netherlands) for 7 days, three times daily. The participants were instructed not to wear their conventional denture for 2 weeks and rinse their mouth with 0.2% chlorhexidine (Corsodyl, GlaxoSmithKline, Utrecht, the Netherlands). After 2 weeks, the sutures were removed and the conventional denture was relined with a soft reline (Soft-Liner, GC, Leuven, Belgium). After 3 months of osseointegration the implants were provided with healing abutments during a second surgical procedure, which was followed by the prosthetic procedure.

### Prosthetic procedure

Using a stock metal tray (Schreinemakers; Clan Dental Products, Maarheeze, the Netherlands), a preliminary alginate impression (Cavex CA 37; Cavex Holland BV, Haarlem, the Netherlands) was made to enable the dental technician to fabricate an individual impression tray of acrylic resin (Lightplast base plates; Dreve Dentamid GmbH, Unna, Germany). After relining the rims of the individual tray with a thermoplastic material (ISO Functional; GC Europe A.G., Leuven, Belgium) and placing screw-retained impression copings to the implants, the final impression was made with a polyether material (Impregum F; 3 M ESPE, St. Paul, MN, USA). The vertical dimensions and intermaxillary relations were recorded with wax rims and a pin registration device. Acrylic resin teeth (Ivoclar SR Orthotyp DCL and Ivoclar VivodentPE, Ivoclar Vivadent AG, Schaan, Liechtenstein) were positioned for a trial arrangement following a bilateral balanced occlusion concept.

**Fig. 2:**
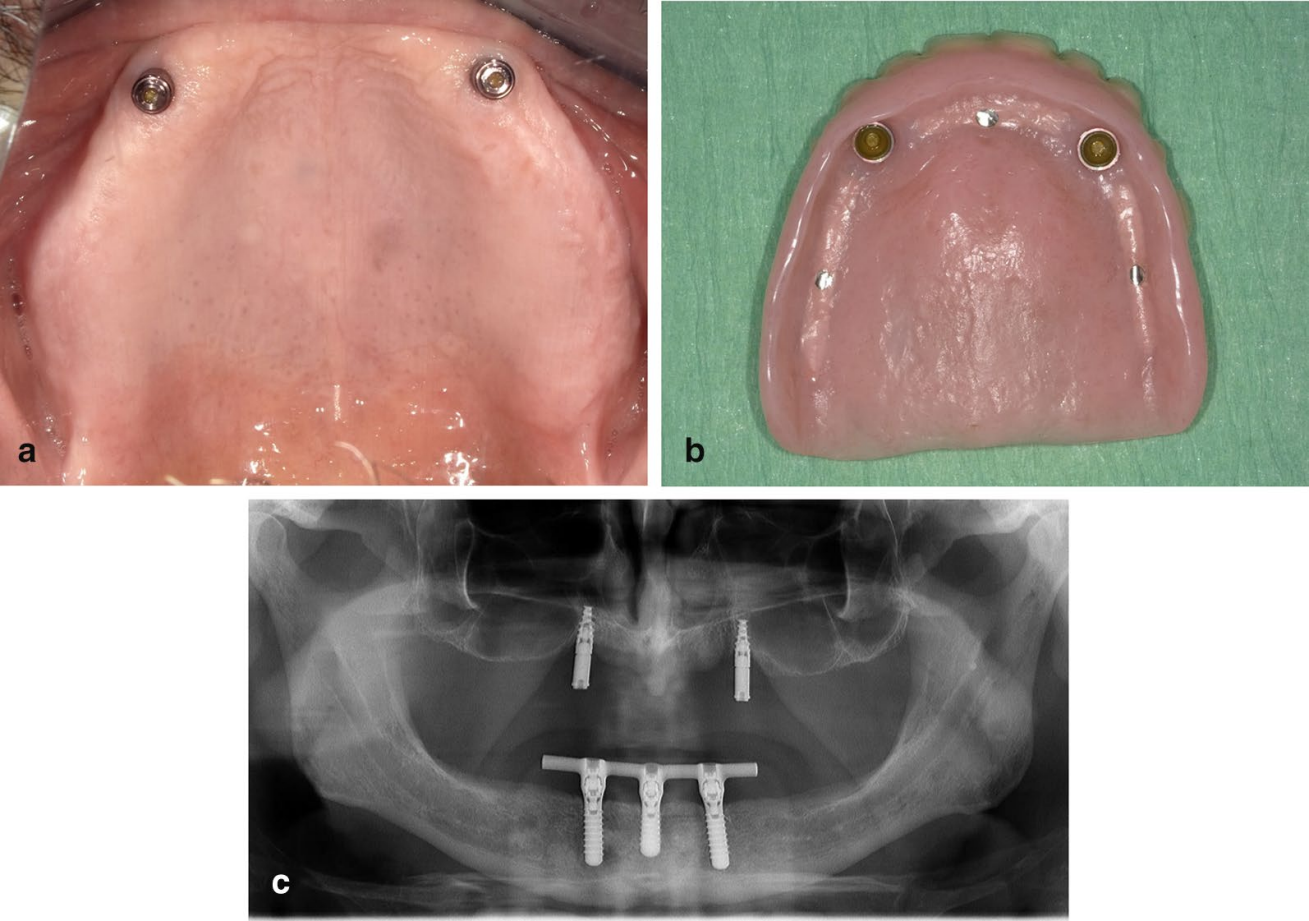
**a** Patient with a maxillary IOD after 12 months. **b** Overdenture of the same patient. **c** Panoramic radiograph of the same patient

For the implant superstructure two matrix copings (Locator® RTX, Zest Dental Solutions, Carlsbad, California, USA) were placed into the overdenture’s base. All copings were initially provided with medium force nylon attachment caps, enabling up- or down-grading the retentive force if needed. For additional support, all the overdenture were designed with full palatal coverage (Fig. 2a–c). At overdenture placement, all the participants received hygiene instructions for their overdenture and superstructure and were scheduled for routine maintenance recalls. All prosthetic procedures were accomplished by one prosthodontists (HJAM).

### Outcome measures

The study’s primary outcome measures were implant and overdenture survival. Secondary outcome measures were MBLC, clinical outcome measures (presence of plaque and calculus, mucosal health, bleeding on probing and PDC), masticatory performance, patient-related outcome measures (PROMs) and complications. Implant and overdenture survival, MBLC and clinical outcome measures were evaluated after 1 month (T1) and 12 months (T12) after placement of the prosthesis. Complications were recorded throughout the whole follow-up period. Masticatory performance and PROMs were evaluated prior to treatment (T0) and at T12.

### Implant and overdenture survival

Implant survival was defined as the percentage of initially placed implants still present and immobile at T12. Implant mobility was assessed by performing a percussion test. Overdenture survival was defined as the percentage of overdentures still present at the follow-up evaluation.

### Marginal bone level change

Intra-oral radiographs were made at T1 and T12 to assess the MBLC. The radiographs were standardised with the paralleling extension-cone technique using a corresponding system (RINN, Dentsply, Elgin, IL, USA). The solitary attachments were digitally cropped from the digital radiographs, aiding in blinded assessment. Using computer software (Biomedical Engineering, UMCG, the Netherlands) the marginal bone level change was then assessed, utilising the implant diameter (3.5 mm) for measurement calibration and using the neck of the implant as a horizontal reference line. Measurements were done by one examiner (HJAM) at the mesial and distal side of the implant and from the neck of the implant to the crestal bone, perpendicular to the reference line. This measurement method was evaluated by Telleman et al. [13] with a Cronbach’s alpha of 0.867 which is similar to an almost perfect agreement. MBLC was defined as the difference in bone height between the measurements made at T1 and T12.

### Clinical outcome measures

Measurements were done by one examiner (PO). Probing depth (PD) was measured the mesial, vestibular, distal and oral site of each implant using a manual periodontal probe. The distance between the marginal border of the mucosa and the tip of the periodontal probe was noted as probing depth. PDC was calculated by subtracting the measurements of T1 from T12.

The presence of plaque was assessed using the index described by Loë and Silness [14], ranging from 0 to 3, corresponding with no plaque detection (0); plaque accumulation after probing (1); visible plaque detection (2); and an abundance of visible plaque (3).

The presence of calculus was scored 0 or 1, corresponding with the absence (0) or presence of calculus (1), respectively.

The health of the peri-implant mucosa was assessed using the modified Löe and Silness index [14], ranging from 0 to 3, corresponding with normal mucosa (0); mild inflammation with slight oedema and redness (1), moderate inflammation with oedema, redness and glazing (2); and severe inflammation with marked redness, oedema and ulceration (3).

Bleeding on probing was assessed using the Mombelli et al. index [15], ranging from 0 to 3, corresponding with no bleeding (0); isolated bleeding (1); confluent bleeding along the mucosal margin (2); and heavy or profuse bleeding (3).

### Masticatory performance

To objectively measure masticatory performance, the mixing ability test (MAT) was performed. For the MAT each participant was asked to chew 20 strokes on a prefabricated paraffine wax tablet with a red and blue layer. Chewing the tablet gradually decreases the spread of blue and red colour intensities, representing the masticatory performance. To prepare the chewed tablet for analysis, it was heated to 28 °C and compressed to a thickness of 2.0 mm using a hydraulic hand press at 50 bar. Both sides of each tablet were then optically scanned using a high quality scanner (Epson V750, Long Beach, California), resulting in an image with a spread of blue and red colours [16]. Using computer software (Adobe Photoshop CS3; Adobe, San Jose, California) the mixing ability index (MAI) was obtained by measuring the intensity distributions of the red and blue colours of the combined images [17]. The MAI ranges from 30 (badly mixed) to 5 (a theoretically perfect mix).

### Patient-related outcome measures

The PROMs were assessed using validated questionnaires, i.e., the chewing ability questionnaire (CAQ) [18]. the denture complaints questionnaire (DCQ) [19] and the Dutch version of the Oral Health Impact Profile 49 questionnaire (OHIP-49NL) [20]. The CAQ was used to test masticatory ability. The participants were asked to rate their ability to chew nine different foods on a three-point scale, e.g., good, moderately, or bad. The foods were divided in three categories, e.g., soft foods (boiled vegetables, crustless bread, minced meat), tough foods (crusty bread, steak, Gouda cheese), and hard foods (apple, carrot, peanuts). The DCQ consists of 54 questions, divided in six categories, addressing functional problems of the lower denture, the upper denture, general functional complaints, denture aesthetics, facial aesthetics, and accidental lip, cheek, and tongue biting (‘neutral space’). Questions could be answered on a four-point scale, ranging from 0 (no complaints) to 3 (severe complaints). At the end of the questionnaire, the participant is asked to rate the overall denture satisfaction on a ten-point scale, ranging from 1 (very bad) to 10 (excellent). The OHIP-49NL questionnaire consists of 49 questions, divided in seven categories, i.e., functional limitation, physical pain, psychological discomfort, physical disability, psychological disability, social disability and handicap. Questions could be answered on a five-point scale, ranging from 0 (never) to 4 (very often).

### Complications

Complications were scored throughout the whole follow-up period. Examples of complications were loosening or fracture of denture teeth, replacement of nylon caps, and adaptation of the denture edges because of pressure ulcers.

### Statistical analysis

Implant and overdenture survival, MBLC, clinical parameters and complications were presented as descriptive statistics. Continuous data (MAI and PROMs) were tested for normality using the Shapiro Wilk test and analysing Q–Q-plots. In case normality could be assumed, these data were further analysed using the paired samples *t* test, if not, than the Wilcoxon Matched Pairs Signed Ranks test was used as a non-parametric alternative. A *p* value of < 0.05 was considered statistically significant. All analyses were performed with SPSS 23.0 software (SPSS, Inc, Chicago, Illinois).

## Results

### Patient characteristics

**Fig. 3:**
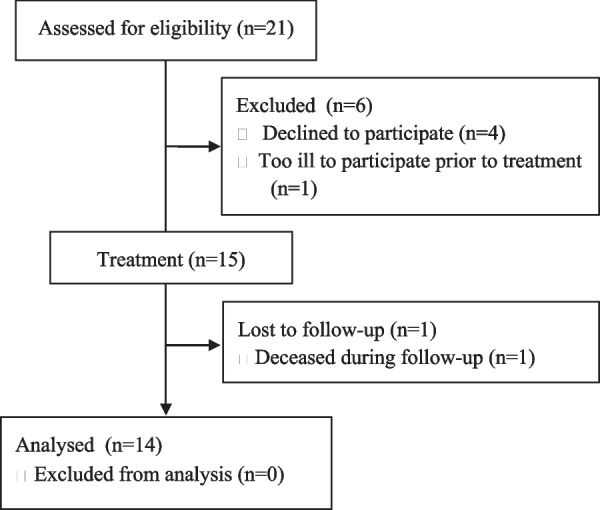
Follow-up flow diagram

For this study, 15 participants with a mean age of 66.7 ± 7.9 years (range: 48–80 years; 8 male and 7 female) were included. All the participants’ were either dentate or had an IOD in the mandible. One participant deceased before the 12-month evaluation, resulting in a total of 14 participants available for the final analysis (Fig. 3).

### Implant and overdenture survival rate

**Fig. 4:**
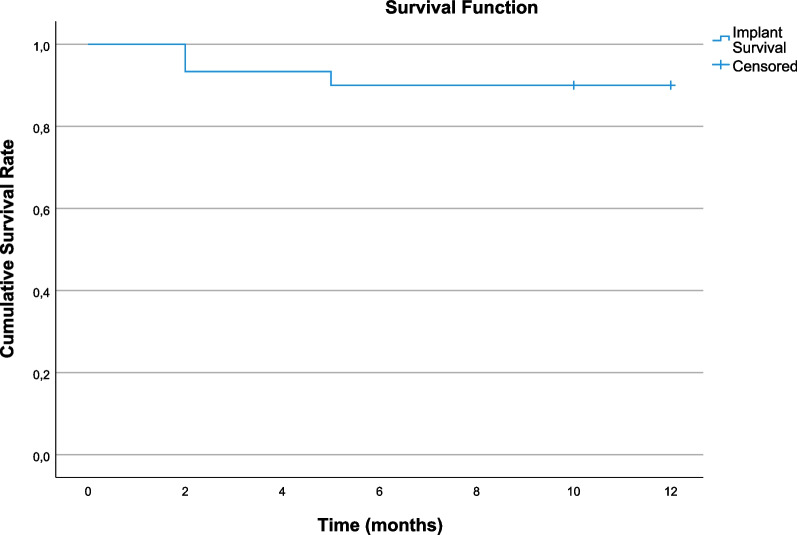
Implant survival rate

**Fig. 5:**
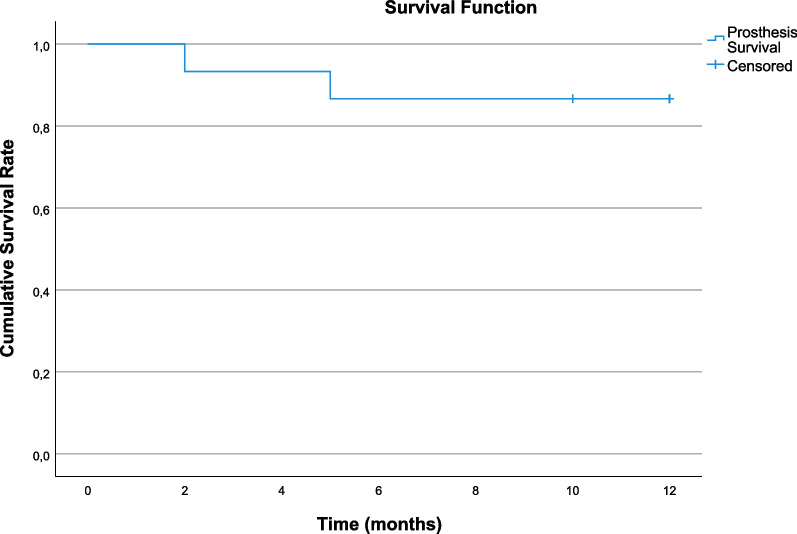
Prosthesis survival rate

During the osseointegration phase no implants were lost. During the first year of function, one participant lost both implants and another participant lost one implant due to loss of osseointegration (Table 1). As a consequence, prostheses of both participants were also lost, since both prostheses lacked retention after losing the implants, resulting in an implant survival rate of 89.3% (Fig. 4) and an overdenture survival rate of 85.7% (Fig. 5).

**Table 1:** Implant and overdenture survival, radiographical and clinical parameters

	T1	T12
Participants (*n*)	15	14
Implants survival (*n* (%))	30 (100%)	25 (89.3%)
Prostheses survival (*n* (%))	15 (100%)	12 (85.7%)
Mean marginal bone level change (SD)	NA	− 0.5 mm (0.7)
Mean probing depth change (SD)	NA	0.0 mm (1.0)
Median plaque-index [Q1–Q3]	0.5 [0–1]	0 [0–1]
Median calculus-index [Q1–Q3]	0 [0–0]	0 [0–0]
Median gingival-index [Q1–Q3]	0 [0–1]	0 [0–0]
Median bleeding index [Q1–Q3]	0 [0–1]	0 [0–1]

*Q1–Q3*  interquartile range, *mm*  millimetres, *n* = number of, *NA*  not applicable, *SD*  standard deviation

Due to his impaired general health, the participant who lost two implants chose to be further treated with a conventional denture. The participant who lost one implant was reimplanted. After successful osseointegration a new IOD was fabricated.

### Marginal bone level change and clinical outcomes

The remaining 12 participants completed the 1-year follow-up. Mean MBLC after 12 months of function was − 0.5 ± 0.7 mm. Mean PDC was 0.0 ± 1.0 mm. Other clinical outcomes were low at T1 and remained low at T12 (Table 1).

### Masticatory performance and PROMs

Data on masticatory performance (MAI) and PROMs are listed in Table 2. For MAI, the Shapiro–Wilk test did not result in a significant difference and the data sets followed a normal distribution in the Q–Q-plots, and therefore, normality of data was assumed. At T0, the mean MAI was 22.1 ± 2.5. At T12, the MAI significantly improved (18.7 ± 2.4, *p* = 0.002, Paired-samples *t* test). For the PROMs the Shapiro–Wilk resulted in a significant difference, and therefore, normality was not assumed. For the CAQ all items improved significantly between pre-treatment and T12, favouring IOD treatment. For the DCQ and the OHIP-NL49 most items improved significantly between pre-treatment and T12, favouring IOD treatment, with the exception of “facial aesthetics” and “neutral space” in the DCQ.

**Table 2:** Within-group comparison of masticatory performance and PROMs, before treatment (T0) and 12 months after treatment

	T0	T12	*p* value
*Masticatory performance*			
Mean Mixing Ability Index (SD)	22.1 (2.5)	18.7 (2.4)	0.002^§,^**
*Chewing ability questionnaire*			
Median total food chewing score [Q1–Q3] (max. 18)	12.5 [12;13.75]	4 [2.25;10]	0.005^†,^**
Median Soft foods score [Q1–Q3] (max. 6)	2 [1;3]	0 [0;0.75]	0.011^†,^*
Median tough foods score [Q1–Q3] (max. 6)	5 [4;6]	0.5 [0;3.5]	0.008^†,^**
Median hard foods score [Q1–Q3] (max. 6)	6 [5.25;6]	4 [2;5]	0.007^†,^**
*Denture complaints questionnaire*			
Median functional complaints upper denture [Q1–Q3] (max. 27)	16 [14–21]	2 [0–6]	0.003^†,^**
Median Functional complaints in general [Q1–Q3] (max. 54)	22 [17–31]	1 [0–3]	0.003^†,^**
Median Facial aesthetics [Q1–Q3] (max. 9)	0 [2–4]	3 [3–9]	0.092^†^
Median “Neutral Space” [Q1–Q3] (max. 9)	1 [1–3]	1 [0–2]	0.058^†^
Median Aesthetics [Q1–Q3] (max. 36)	0 [2–9]	0 [0–1]	0.028^†,^*
Median General satisfaction score upper denture [Q1–Q3]	3 [1–4]	8 [6–9]	0.003^†,^**
*Oral health impact profile-NL49*			
Median Functional limitation [Q1–Q3] (max. 36)	20.5 [18–26.5]	4 [0.8–8.8]	0.002^†,^**
Median Physical pain [Q1–Q3] (max. 36)	16.5 [14.25–22.25]	8 [4.5–11.5]	0.003^†,^**
Median psychological discomfort [Q1–Q3] (max. 20)	14.0 [7.75–18.25]	4.5 [2.3–8.3]	0.002^†,^**
Median physical disability [Q1–Q3] (max. 36)	19.5 [15.5–22.75]	3.5 [2.3–9.3]	0.002^†,^**
Median psychological disability [Q1–Q3] (max. 24)	11.0 [5.8–16.5]	2.5 [0–8]	0.002^†,^**
Median social disability [Q1–Q3] (max. 20)	8 [0.3–10.8]	2 [1–5.8]	0.044^†,^*
Median handicap [Q1–Q3] (max. 24)	6.5 [1.5–11.8]	3 [0–4.8]	0.011^†,^*
Median total OHIP-NL49 score [Q1–Q3] (max. 196)	71.5 [58.5–105]	16.5 [9.3–48.3]	0.002^†,^**

Q1–Q3: interquartile range; max. = maximal score

*p < 0.05, **p < 0.01, ^**§**^Paired-Samples *T* test, ^†^Wilcoxon Matched Pairs Signed Ranks test

### Complications

All implants were placed without any peri-operative complications. The wound healing was uneventful, although two patients suffered from severe haemorrhage without additional bleeding intra-orally 1 day after implant placement. Both patients regularly used direct-acting oral anticoagulants. In one patient the nylon caps had to be replaced due to wear and in three patients one abutment were retightened.

## Discussion

When considering implant treatment in elderly and medically compromised patients, a holistic approach is required considering treatment risks, costs and burden related to the patients general health versus functional and psychosocial benefits of treatment [21]. With this case series we intended to lower the risks and burdens when treating patients with an atrophic edentulous maxilla, enabling the treatment of patients that were otherwise excluded. Enabling treatment without general anaesthesia and extensive bone augmentation, treatment costs and time are reduced, and potential cognitive and physical risks and burdens associated with these treatments are prevented [6, 22, 23].

Based on the findings of the present case series, patients that are treated with a 2-IOD retained by solitary attachments have satisfactory radiographical and clinical outcomes and improved patient-related and masticatory outcomes 12 months after treatment, but do have a relatively higher risk of losing an implant due to loss of osseointegration, compared to most other studies on maxillary IODs, ranging from 95% to 100% after up to 5 years [2–4, 9–11]. Some studies do report survival rates comparable to the present study [8, 24]. Implant overload is suggested as a possible cause for the loss of osseointegration in 2-IOD-implant maxillary overdentures [10]. To anticipate possible overload, we used palatal coverage and a resilient system to connect the implants to the overdenture. In vitro research has shown that both these factors can reduce the load on the implants [25, 26]. However, overload is still controversial in implant research. Low bone volumes, which was the case in all the participants, has been recognised as an important risk factor for loss of osseointegration [26, 27] and, therefore, may have been the main reason for the implant failure in the present study.

### Marginal bone level change and clinical parameters

The mean marginal bone level change in the present study is well within the range of bone remodelling that can be expected within the first year [29]. This is also comparable to other studies, reporting a MBLC after the first year of − 0.4 mm to − 0.7 mm for 2/3-IODs [8–11] and − 0.3 to − 0.6 for 4-IODs [2, 5, 30–32], regardless of connection type (bars or solitary attachments). Clinical parameters in the present study were favourable throughout the entire study period. Clinical parameters were not reported in the other 2/3-IOD studies [8–11], but were in line with the 4-IOD studies [2, 5, 30–32]. Therefore, it is assumed that these clinical parameters did not contribute to the relatively low implant survival rate in the present study.

### Improved mastication

Both the masticatory ability (CAQ) and performance (MAI) improved significantly at T12 compared to pre-treatment. A study comparing 4-IODs with bar and solitary retention analysed both parameters with similar tests [5]. Interestingly, the pre-treatment scores of the present study were less favourable (CAQ total score 12.5 versus 9 for bars and 8 for solitary attachments; MAI 22.0 versus 20.2 for bars and 20.5 for solitary retention), but at T12 the scores of the present study and solitary attachments in the 4-IOD study are comparable (CAQ total score 4 versus 4; MAI 18.7 ± 2.4 versus 18.0 ± 1.7). Specifically hard foods seem relatively harder to chew when using solitary attachments. The greater leverage caused by biting off hard foods anteriorly, such as apples or carrots, may cause the solitary attachments to dislodge more easily compared to bar attachments, which could explain these results. Nonetheless, the improvement in both subjective and objective parameters suggests an independence over the number of implants used to retain an IOD in the maxilla.

### Improved PROMs

Four studies reporting on 2/3-IODs also reported PROMs and showed high satisfaction scores throughout the entire study period [8–11]. The questionnaires differed to the present study’s, hindering proper comparison. A study reporting on 4-IODs retained with bars or solitary attachments used the same questionnaires as in the present study [32]. Scores on OHIP-NL49 and DCQ were favourable and similar to the present study. A similar result was reported by a study researching mandibular one- and two-implant overdentures with ball-attachments [33]. After 1, 3 and 5 years, the participants’ mean satisfaction improved significantly for both groups, but did not differ between groups. These results suggest that improvement of patient satisfaction may be independent of the number of implants or the type of retention used to retain an overdenture.

### Strengths and limitations

Though more invasive compared to the present therapy, reconstructive surgery prior to implant placement is a safe and reliable treatment, and therefore, most patients are willing to be treated this way. Therefore, an alternative approach such as described in the present study is not offered to the patient very often, which limited the group size even after 3 years of inclusion, which may have affected the power of the present study’s results. In addition, the follow-up of this case series is short, limiting the results on complications that may increase in the long term. Nevertheless, in our opinion the present study gives a complete overview of the risks and benefits of 2-IOD treatment in patients unwilling to be treated with reconstructive maxillary surgery prior to maxillary IOD treatment.

### Future research

Four implants should still be considered the gold standard for maxillary overdenture therapy. However, to be able to offer customised care for any patient, future research should continue to focus on alternative therapies such as presented in the present study.

## Conclusions

Within the limitations of this study, it can be concluded that patients with extreme resorption of the maxilla that are unwilling to be treated with reconstructive surgery, benefit from two-implant maxillary overdentures retained by solitary attachments in terms of improved masticatory functioning and denture satisfaction, but with a relatively high risk of implant loss.

## Data Availability

The data sets used and/or analysed during the current study are available from the corresponding author on reasonable request.

## Abbreviations

IOD: Implant overdenture
MBLC: Marginal bone level change
PDC: Pocket depth change
CBCT: Cone beam computed tomography
ASA: American Society of Anesthesiologists score
PROMs: Patient-related outcome measures
MAT: Mixing ability test
MAI: Mixing ability index
DCQ: Denture complaints questionnaire
CAQ: Chewing ability questionnaire
OHIP-49NL: Dutch version of the Oral Health Impact Profile-49 questionnaire
